# Nutritional Interventions for Enhancing Female Fertility: A Comprehensive Review of Micronutrients and Their Impact

**DOI:** 10.1155/nrp/2137328

**Published:** 2025-08-05

**Authors:** Faezeh Mashhadi, Zahra Sedghi, Ava Hemmat, Raha Rivaz, Fatemeh Roudi

**Affiliations:** ^1^Department of Nutrition, Faculty of Medicine, Mashhad University of Medical Sciences, Mashhad, Iran; ^2^Metabolic Syndrome Research Center, Mashhad University of Medical Sciences, Mashhad, Iran

## Abstract

Infertility significantly impacts individuals and society, necessitating effective strategies for its management. Among the various factors influencing female fertility, micronutrients play a crucial role in reproductive health by supporting oocyte quality, hormonal balance, and implantation processes. This narrative review examines the importance of optimal preconception micronutrient intake in enhancing female fertility. By analyzing research from various scientific databases, including PubMed, Google Scholar, and ScienceDirect, spanning from 2000 to April 2024, we highlight the impact of key micronutrients such as folate, vitamin D, iron, selenium, and antioxidants on fertility outcomes. Deficiencies in these nutrients have been associated with impaired ovarian function, disrupted menstrual cycles, and increased risk of pregnancy complications. Given the prevalence of micronutrient inadequacies among women of reproductive age, this review underscores the need for evidence-based nutritional interventions and standardized supplementation guidelines. The findings aim to bridge the gap between research and clinical practice, providing healthcare professionals with insights to optimize fertility care through targeted nutritional strategies.

## 1. Introduction

Infertility presents a significant global health concern, affecting 20%–30% of women of reproductive age worldwide. The World Health Organization (WHO) characterizes infertility as the inability to conceive after 12 months of unprotected sexual intercourse [1]. Over the past decade, considerable attention has been directed toward exploring the relationship between dietary patterns and human fertility [2] particularly in light of declining fertility rates in industrialized nations, which have reached historically low levels. This underscores the urgency of identifying modifiable factors, such as diet, that influence fertility outcomes, thereby holding substantial clinical and public health significance [3].

Various lifestyle-related factors, including poor preconception nutrition, obesity, anxiety, and stress, have consistently been identified as significant contributors to infertility and adverse fertility outcomes. Among these, preconception diet stands out as a key modifiable risk factor, yet many women fail to meet optimal nutritional intake during this critical period. As a result, increasing research has focused on understanding the role of diet in reproductive health [4–6].

Micronutrient status represents another critical modifiable factor that may impact female fertility, given the essential roles of vitamins and minerals in various physiological processes. Adequate levels of these micronutrients are essential for oocyte quality, maturation, fertilization, and implantation. Additionally, antioxidants help counteract oxidative stress (OS), a well-documented factor contributing to infertility. Studies have reported suboptimal levels of certain micronutrients in infertile women, as well as in a subset of women of childbearing age, potentially impairing their ability to conceive [3].

Despite the growing recognition of the association between micronutrients and reproductive outcomes in women, official guidelines for preconception micronutrient supplementation remain limited. Therefore, synthesizing pertinent evidence and elucidating key components in this domain is imperative for advancing knowledge and informing evidence-based nutritional recommendations for couples planning pregnancy and their healthcare providers. This comprehensive review aims to consolidate scientific insights into the role of micronutrients in women's fertility.

## 2. Materials and Methods

The information presented in this narrative review was compiled from scholarly articles retrieved from various reputable online databases, including PubMed, Google Scholar, and ScienceDirect, along with publications and databases of governmental organizations. Our search spanned from the year 2000 to April 2024, aiming to comprehensively cover relevant literature. A summary of our search strategy is provided in Table 1.

### 2.1. L-Arginine and Female Reproductive Health

L-Arginine, a semiessential amino acid, plays a crucial role in reproductive health and performance [7]. Since the discovery of the L-arginine–nitric oxide (NO) system, its physiological and clinical implications have been extensively studied [8]. Acting as an antioxidant, L-arginine helps protect against OS and enhances NO production, improving uterine blood flow in women [9–11]. L-Arginine enhances NO production, which plays a critical role in regulating uterine blood flow. Defective NO synthesis has been associated with various adverse reproductive outcomes. Studies have shown that inhibiting NO can lead to a significant (60%) reduction in uterine artery blood flow, while NO donors improve subendometrial perfusion. These findings suggest that NO primarily acts to optimize uterine blood flow rather than causing pathological increases in uterine pressure, supporting its beneficial role in reproductive health [12].

Studies show that L-arginine supplementation positively influences pregnancy outcomes and mitigates complications. For example, So et al. reported a significant reduction in pregnancy-induced hypertension (PIH) among high-risk patients receiving L-arginine compared to those who did not [10, 13].

Additionally, Battaglia et al. found that women classified as poor responders to infertility treatment experienced an increased oocyte yield and a potential improvement in pregnancy rates with oral L-arginine supplementation [11]. Despite these benefits, the optimal daily dosage for women trying to conceive remains undetermined. Takasaki et al. noted endometrial-proliferative effects at a dosage of 6 g/day, while Battaglia et al. reported enhanced ovarian function at 16 g/day [14].

L-Arginine is naturally present in various food sources, including seafood, nuts, seeds, meats, and soy proteins, with an average dietary intake of about 5 g/day. Popolo et al. reported significant increases in serum arginine levels following oral supplementation [15, 16]. The NO pathway, regulated by L-arginine, plays a crucial role in ovarian function. Oral supplementation during ovarian stimulation has been associated with increased levels of L-arginine, L-citrulline, and nitrite/nitrate in plasma and follicular fluid, which in turn improves perifollicular artery blood flow [11, 17].

The L-arginine-NO system is essential at all stages of female reproduction, and imbalances can negatively impact in vitro fertilization (IVF) outcomes [8]. Arginase, which competes with NO synthase for L-arginine, may reduce its bioavailability, affecting reproductive processes. Poor responders to IVF treatment with L-arginine exhibited higher plasma and follicular fluid levels of arginine, citrulline, and NO_2_/NO_3_, leading to improved outcomes [18]. Moreover, L-arginine supplementation can enhance endometrial receptivity and reduce risks of fetal loss, intrauterine growth restriction, and preeclampsia [8, 19, 20]. However, during controlled ovarian hyperstimulation cycles, L-arginine may negatively affect embryo quality and pregnancy rates due to inconsistent follicular growth [21].

Furthermore, L-arginine shows potential in improving oocyte quality and development in vitro, offering alternative therapies for endometriosis-associated subfertility/infertility [22].

### 2.2. Omega 3 Fatty Acids and Female Reproductive Health

Omega-3 fatty acids, found in nuts and fish, are being studied for their potential impact on female fertility [23, 24]. Research indicates that omega-3 supplementation may enhance folliculogenesis, oocyte maturation, embryo quality, and implantation by influencing prostaglandin biosynthesis in the ovary and endometrium. Stanhiser et al. reported higher conception rates among women supplementing with omega-3 compared to nonsupplementers [25].

Omega-3 intake has also been associated with increased pregnancy rates following IVF [26]. Docosahexaenoic acid (DHA), the most abundant omega-3 PUFA in follicular fluid, has been linked to better reproductive outcomes [24, 27].

However, studies on the relationship between omega-3 PUFA levels and assisted reproductive technology (ART) outcomes are mixed. Some show positive correlations with clinical pregnancy and live birth rates (LBRs), while others do not, possibly due to differences in body mass index (BMI) among study populations [28, 29].

Omega-3 PUFAs are essential for oocyte membrane composition and successful fertilization. Lower omega-3 intake is linked to reduced fecundability and higher estradiol levels, while higher DHA intake is associated with lower anovulation risk and increased estradiol levels [24, 30–32].

These fatty acids also promote the production of anti-inflammatory prostaglandins, which are beneficial for reproductive function [32–35]. Although some studies suggest omega-3 intake improves IVF success and reduces pregnancy loss, the evidence remains inconclusive due to conflicting data [36–39].

In summary, a high intake and elevated serum levels of omega-3 PUFAs may positively influence ART outcomes and embryo quality; however, further research is required to clarify their precise role in female fertility.

### 2.3. L-Carnitine and Female Reproductive Health

L-Carnitine, a derivative of lysine, and its acetylated form, acetyl L-carnitine (ALC), play crucial roles in maintaining the oxidative and metabolic health of the female reproductive system [40, 41]. Edris and Barakat reported that daily supplementation with 3 g of L-carnitine significantly improved endometrial receptivity, implantation rates, clinical pregnancy rates (CPRs), and LBRs in women with prior implantation failures during intracytoplasmic sperm injection (ICSI)/FET cycles [42].

L-Carnitine functions as an intramitochondrial transporter for acyl groups, facilitating fatty acid oxidation [43]. Its potent antioxidant properties, combined with its minimal side effects, make it a promising therapeutic option for female infertility associated with OS, a factor that impairs oocyte lipid peroxidation (LPO), fertilization, and embryo development [41, 44–46].

ALC enhances reproductive functions through its antioxidative properties [43]. It plays a role in intermediary metabolism, facilitates fatty acid transport during beta-oxidation, and may influence the hypothalamic–pituitary–gonadal (HPG) axis, thereby impacting female reproductive function [41, 47, 48].

Agarwal et al. suggest that L-carnitine and ALC enhance female fertility by boosting energy production in oocytes and shielding reproductive cells from oxidative damage. These compounds may also improve serum hormone levels and regulate the HPG axis, making them potential reproductive biomedicines and fertility enhancers [41]. The primary dietary sources of L-carnitine include meat, fish, and other animal products such as milk [49].

### 2.4. *N*-Acetylcysteine and Female Reproductive Health

*N*-Acetylcysteine (NAC), known for its potent antioxidant properties, is effective in treating various diseases, including reproductive system disorders. With rising environmental pollution affecting fertility, NAC shows promise in enhancing human fertility health [50].

As a precursor to glutathione (GSH), NAC's antioxidant activity involves scavenging free radicals and improving oocyte quality, especially in aged mice undergoing oocyte cryopreservation [51].

NAC supplementation helps upregulate GSH levels, counteracting OS, and stabilizes reactive oxygen species (ROS) by donating electrons from its sulfur group. Additionally, NAC promotes NO production, aiding arterial dilation and offering therapeutic benefits [50].

Studies show NAC's ability to regulate luteinizing hormone (LH), follicle-stimulating hormone (FSH), and testosterone levels against various toxins [52, 53]. By preventing the increase of OS markers, NAC strengthens the antioxidant defense system [50, 54, 55]. A research found higher pregnancy [56] rates with NAC supplementation, likely due to improved oocyte quality and reduced COX-2 expression [56].

A proinflammatory and ROS-dominated environment can damage gametic DNA, leading to embryo fragmentation, implantation failure, and placental abnormalities, increasing miscarriage rates (MRs) [57]. NAC's mucolytic effects also improve cervical mucus quality, facilitating sperm movement and enhancing tubal function [58, 59].

A meta-analysis indicated NAC's efficacy as an adjuvant in polycystic ovary syndrome (PCOS)-related and unexplained female infertility, particularly in women with high BMI, insulin resistance, and OS. Supplementation from Day 3 to Day 7 at dosages of 1200 or 1800 mg per day has shown promising results [57].

In conclusion, NAC shows promise for treating female infertility due to its antioxidant and hormone-regulating properties. However, further research is required to fully understand its impact on fertility treatment. Cysteine, a precursor to NAC, is present in most high-protein foods, including chicken, turkey, yogurt, cheese, eggs, sunflower seeds, and legumes.

### 2.5. Coenzyme Q10 and Female Reproductive Health

Coenzyme Q10 (CoQ10) is a lipid-soluble quinone that functions as an antioxidant and supports ATP synthesis [60]. Supplementation with CoQ10 has demonstrated potential in improving CPRs; however, evidence regarding its impact on LBRs and MRs remains limited [61].

CoQ10 is crucial for mitochondrial activity, especially in energy-demanding tissues [62, 63]. Age-related declines in CoQ10 levels, which become evident after the age of 30, may contribute to reduced fertility and an increased incidence of embryo aneuploidy. Low CoQ10 levels have been associated with spontaneous abortion and are correlated with oocyte maturation and embryo quality in IVF cycles [64].

Meta-analyses suggest CoQ10 supplementation increases CPR in women undergoing ART treatments. While its effects on LBR and MR are similar to placebo, CoQ10 benefits women with PCOS and those undergoing IVF-ICSI [65, 66].

Despite its potential benefits, evidence supporting CoQ10's role in fertility remains inconsistent, largely due to variations in treatment protocols, dosage, and study designs. Nevertheless, CoQ10 may enhance gamete physiology, support embryo development, and improve pregnancy outcomes by mitigating OS. It may also help counteract age-related fertility decline, although its effects may diminish upon discontinuation [67].

In conclusion, supplementing with CoQ10 presents a low-risk and cost-effective method to enhance reproductive performance, especially under conditions of aging and environmental stress. Nutritional sources rich in CoQ10 include meat, fish, nuts, and certain oils. The suggested daily dosage varies from 5 to 30 mg/kg [62, 68–70].

### 2.6. Beta-Carotene and Female Reproductive Health

β-Carotene, a dietary carotenoid and precursor of vitamin A, plays a crucial role in reproductive health. Daily supplementation enhances its bioavailability in oocyte microenvironments, potentially improving follicular development and oocyte quality under OS [71].

Short-term β-carotene intake positively influences ovarian steroidogenesis, increasing progesterone synthesis and ovarian activity [72, 73]. It also acts as a potent antioxidant, reducing lipid oxidation reactions and alleviating OS-induced inhibition of oocyte development and maturation [73].

Higher β-carotene concentrations are associated with shorter time to pregnancy, especially in overweight/obese women and younger age groups [74–76]. This effect may be attributed to the antioxidant properties of carotenoids, which help neutralize ROS generated during ovulation. Granulosa cells exhibit high metabolic activity, and the follicular environment contains numerous macrophages and neutrophils, contributing to OS. By scavenging free radicals, reducing DNA damage, and preventing LPO, carotenoids may support follicular health and improve fertility outcomes [77, 78].

Supplementation improves oocyte quality and ovarian function, reducing lipid oxidation, ROS production, and early apoptotic cell rates. β-Carotene enhances cytoplasmic maturation of oocytes during the second meiotic division, observed in bovine models [74]. While specific biological mechanisms are unclear, its antioxidant properties and potential hormonal interactions suggest promising implications for reproductive function [75].

Further research is needed to understand the clinical significance of β-carotene supplementation in improving fertility outcomes in women attempting pregnancy.

### 2.7. Vitamin D and Female Reproductive Health

Vitamin D, an essential hormone and fat-soluble steroid, regulates calcium and phosphorus metabolism [79]. It plays a crucial role in female reproduction by regulating gene expression in reproductive tissues, preventing cell proliferation, and stimulating differentiation. Studies show a link between vitamin D deficiency (VDD) and fertility disorders like PCOS and pregnancy-related issues. Lata et al. found a concerning 64.28% prevalence of VDD among infertile women [80]. Adequate vitamin D consumption, alongside other nutrients, can improve fertility outcomes but may not prevent abortion in infertile women. Supplementation increases the likelihood of successful fertility outcomes [81]. Vitamin D supplements can enhance pregnancy rates by improving PCOS symptoms, menstruation, follicular growth, and weight loss in obese patients with PCOS and VDD [82]. Vitamin D's effect on calcium homeostasis improves oocyte activation, maturation, menstrual cycle regulation, ovulation, and IVF outcomes [83]. Supplementation improves ovulation and LBRs, reduces miscarriage in women with PCOS, and enhances ART outcomes. Serum or follicular fluid vitamin D levels correlate with IVF and ICSI outcomes, particularly CPRs [84–87].

Obesity reduces vitamin D receptor (VDR) levels, impacting fertility. Weight loss increases VDR and vitamin D levels, improving pregnancy chances [84, 88].

Vitamin D enhances fertility by increasing SIRT1 activity and antioxidant levels [89]. Many foods are good sources of vitamin D, for example, many fish, mushrooms, and cod liver oil, and other food sources such as cheese, beef liver, eggs, dark chocolate, and fortified foods. Since an adequate vitamin D intake of 15 μg per day (by the European Food Safety Authority) is difficult to achieve through diet alone, dietary supplements of vitamin D are usually recommended [90].

Taheri et al. found that daily consumption of 2000 IU of vitamin D in women at risk of deficiency, during prepregnancy, is very useful for preventing complications [91].

The studied articles have a small sample size and high inconsistency, so further investigation of the mechanism of vitamin D treatment in the infertile population is still necessary. Further research is needed due to small sample sizes and high inconsistency in the studied articles regarding vitamin D treatment in the infertile population.

### 2.8. Vitamin E and Female Reproductive Health

Vitamin E is an essential nutrient that plays a crucial role in reproductive health. Its deficiency has been associated with an increased risk of miscarriage, premature birth, preeclampsia, and intrauterine growth restriction. Due to its potent antioxidant properties, vitamin E helps maintain normal female reproductive function by counteracting OS induced by oxygen free radicals [92].

The accumulation of ROS during oogenesis can cause ovarian failure and reduced ovarian reserve. As a cofactor of glutathione peroxidase (GPx), vitamin E plays a critical role in eliminating ROS in the ovary. Studies have shown that vitamin E increases anti-Mullerian hormone (AMH) levels, antral follicle count (AFC), and mean ovarian volume (MOV) in women with occult premature ovarian insufficiency (OPOI) [93].

Vitamin E also reduces OS and may play a role in shortening pregnancy duration [94]. Jargar et al. reported that infertile women exhibit lower levels of alpha-tocopherol and elevated LPO, suggesting that vitamin E is effective in neutralizing free radicals and may contribute to the prevention of female infertility [95].

Vitamin E is present in foods such as almonds, vegetable oils, and grains. Alpha-tocopherol is abundant in olive and sunflower oils, while gamma-tocopherol is found in soybean and corn oils. The recommended daily allowance (RDA) for vitamin E is 15 mg/day for both nonpregnant and pregnant women, with an upper limit (UL) of 1000 mg/day [96].

Despite its known necessity for reproduction, the full extent of vitamin E's beneficial effects on fertility and women's health requires further understanding.

### 2.9. Folic Acid and Female Reproductive Health

Folates are naturally occurring derivatives of folic acid found in food and are essential for DNA synthesis, gametogenesis, fertilization, and pregnancy. Folate deficiency has been associated with an increased risk of anovulation, whereas supplementation has been shown to enhance oocyte quality during IVF, reduce homocysteine levels, and increase folate concentration in follicular fluid, thereby improving pregnancy rates and embryo quality [97].

Folic acid supplementation not only plays a critical role in preventing neural tube defects (NTDs) but also contributes to reducing infertility and improving treatment outcomes. Higher intake of synthetic folate has been linked to a lower likelihood of anovulation and improved ovarian reserve, thereby enhancing fertility potential [98, 99]. Optimal plasma folate levels (6–20 ng/mL) are associated with higher pregnancy and biochemical pregnancy rates, increasing the likelihood of successful implantation [100].

Clinical studies show that folic acid supplements before pregnancy can increase ART success and overall fertility [101]. Hyperhomocysteinemia, often resulting from low folic acid levels, is considered a risk factor for recurrent miscarriage, suggesting that folic acid's role in fertility may be mediated through homocysteine metabolism. A higher folic acid-to-homocysteine ratio has been associated with a 10% reduction in the risk of ovulatory dysfunction [101].

Folic acid intake benefits menstrual cycle function by improving hormonal balance and follicular development. Studies on women undergoing infertility treatment show folate's positive effects on oocyte and embryo quality [99].

Natural dietary sources of folate include spinach, green leafy vegetables, beans, eggs, strawberries, oranges, and kiwi [102]. Most national medical organizations recommend a daily folic acid supplementation of 0.4 mg starting at least 1 month before conception and continuing throughout pregnancy. Women at higher risk are advised to take 1–4 mg daily, beginning one to 3 months before conception, with a UL of 1 mg/day for general supplementation [103]. Further research in diverse populations is needed to explore folate's clinical relevance and biological effects.

### 2.10. Selenium and Female Reproductive Health

Selenium is an essential trace element involved in various physiological processes, particularly as a key component of GPx, an enzyme that protects cell membranes from LPO. Selenium deficiency has been associated with pregnancy complications, miscarriage, and developmental impairments in the fetal nervous and immune systems. Therefore, monitoring selenium levels during the reproductive period is particularly important for women [104].

Selenium and selenoproteins play a crucial role in fertility by contributing to antioxidant balance. The antioxidant activity of the selenium-dependent enzyme GPx within the follicular environment is essential for gametogenesis and fertilization. Studies suggest that lower selenium concentrations in serum and follicular fluid are associated with increased infertility rates [105].

Research by Paskowski et al. found that selenium concentrations in the follicular fluid of women with unexplained infertility were lower compared to those infertile for other reasons, due to Se-binding protein-1, an ovarian autoantibody protein causing premature ovarian failure. Low selenium levels were associated with a 46% higher infertility risk. Functional measures of selenium status, such as GPx and selenoprotein P, are crucial for antioxidant defense, thyroid hormone formation, and DNA synthesis, all affecting fertility [104, 106, 107].

The recommended selenium intake is 40 μg/day for women aged 14 and above before pregnancy, increasing to 60 μg/day during pregnancy.

Although substantial evidence supports selenium's role in female reproduction, the current body of research remains limited. Further studies are needed to reinforce these findings and elucidate the precise mechanisms underlying selenium's impact on fertility.

### 2.11. Nickel and Female Reproductive Health

Nickel, a common component of the earth's crust, is found in food and water from natural and human sources. It usually exists in a bivalent form, its most stable oxidation state [108].

Nickel's bioavailability in humans varies with its solubility, dose, and fasting state, with higher absorption observed when ingested through water on an empty stomach compared to food [34].

In animal studies, nickel exposure has been linked to reproductive issues, including reduced fertility, histopathological changes in reproductive organs, and developmental toxicity. Mice and rats exposed to soluble nickel compounds have shown decreased sperm motility, increased neonatal mortality, and lower neonatal weight. The adverse effects of nickel on reproduction include infertility, abnormalities, and birth defects [34, 108].

The adverse effects of metal ions such as nickel on reproduction and growth are many, which can be mentioned as their effects on male and female infertility or fertility, abnormalities, abortion, and birth defects [109].

In recent years, the effect of metal ions such as nickel on the endocrine glands and disrupting them and the effect on reproductive hormones as well as OS have been introduced as an important mechanism [109].

Nickel's impact on endocrine glands, reproductive hormones, and OS has been identified as a significant mechanism of its reproductive toxicity. However, human data on nickel's reproductive effects are limited, often focusing on male fertility. A recent study indicated a higher spontaneous abortion rate among women in a nickel refinery compared to a control group [109–111].

Research by Kong et al. found that nickel nanoparticles are reproductive toxins in rats, with similar toxic effects observed in both male and female mice. Despite these findings, human studies on the reproductive impact of nickel, especially on women, are scarce. Studies often focus on single metals, overlooking the combined effects of multiple metal exposures and other risk factors on reproductive health [112].

More research is needed to understand the safety standards for nickel exposure and its mechanisms, considering the influence of other metals, factors, and lifestyle on women's reproductive health.

### 2.12. Vitamin A and Female Reproductive Health

Vitamin A, comprising retinal, retinol, and retinoic acids, as well as provitamin A carotenoids, is crucial for reproduction, immune function, and cellular differentiation. It plays a significant role in female reproduction by influencing steroidogenesis, follicular growth, and oocyte and embryo development. Vitamin A acts as an antioxidant, protecting oocyte maturation and embryo development from ROS [113]. ROS, which naturally form during aerobic metabolism, are essential for maintaining oxygen homeostasis during oocyte maturation but can cause OS if not balanced by antioxidants [114].

Antioxidants, including vitamin A, help maintain the oxidant–antioxidant balance in blood and tissues. High antioxidant activity in granulosa cells positively impacts oocyte competence for fertilization, emphasizing the importance of dietary antioxidants [114, 115]. Beta-carotene, a precursor to vitamin A, also protects cells from oxidative damage [114]. Additionally, vitamin A influences inflammatory response, cell differentiation, and embryogenesis. Supplementation before IVF cycles can protect against OS, improve oocyte quality, and shorten time to conception [114, 116].

Vitamin A also promotes ovarian follicular growth and quality, with higher levels in follicular fluid linked to better oocyte quality [114]. The serum vitamin A/total cholesterol ratio inversely correlates with premature ovarian insufficiency (POI) risk, indicating that vitamin A supplementation may help prevent or treat POI [117].

However, excessive vitamin A supplementation can be teratogenic, with doses over 15,000 IU (4500 μg retinol equivalents [RE]) from diet or more than 10,000 IU (3000 RE) from supplements linked to miscarriage and congenital malformations [98]. The UK NICE guidelines recommend avoiding more than 5000 IU (1500 μg) of vitamin A from supplements. Dietary sources of vitamin A include vegetables, dairy products, eggs, and fruits [118].

### 2.13. Vitamin B-6 and Female Reproductive Health

Vitamin B-6, particularly in the form of pyridoxine, plays a significant role in fertility through its impact on homocysteine metabolism [101, 119]. As a coenzyme, it helps convert homocysteine to cysteine, preventing its accumulation, which can slow the transsulfuration process and elevate homocysteine levels [101, 120]. Elevated homocysteine is linked to recurrent miscarriage, impaired chorionic vascularity, trophoblast apoptosis, reduced chorionic gonadotropin levels, and increased endothelial inflammation [101, 121]. High homocysteine in ovarian follicle fluid may also reduce fertilization chances and increase OS, further impacting fertility [101, 122].

Studies show a positive correlation between vitamin B-6 levels and the quality of eggs and embryos [114]. A cohort study found that higher homocysteine levels increased the risk of anovulation by 33%, while a higher folic acid to homocysteine ratio decreased this risk by 10% [101, 123]. Dietary sources of vitamin B-6 include beef liver, tuna, salmon, fortified cereals, chickpeas, poultry, and various vegetables and fruits like dark leafy greens, bananas, papayas, oranges, and cantaloupe. The recommended daily dose of vitamin B-6 increases during pregnancy and lactation from 1.2 mg to 1.9 mg, with a UL of 25 mg [119].

### 2.14. Iodine and Female Reproductive Health

Iodine is essential for thyroid gland function and optimal fertility [101]. A study of 501 women with moderate to severe iodine deficiency showed delayed pregnancy and a 46% decrease in the likelihood of conception per cycle compared to non–iodine-deficient women [124]. Hypothyroidism and thyroid gland antibodies increase the risk of subfertility, miscarriage, and recurrent miscarriage, highlighting the need for adequate iodine for conception and pregnancy progression [125]. Women with low iodine levels experienced prolonged time to conceive, with 28% of the iodine-deficient group failing to conceive within 12 months compared to 12.5% in the iodine-sufficient group [126]. Iodine deficiency is also associated with ovarian cysts, impairing fertility and increasing ovarian cancer risk [126].

Iodine facilitates normal thyroid hormone synthesis and indirectly promotes ovulation by enhancing follicular growth in oocytes through thyroid-stimulating hormone (TSH) acting on FSH receptors [127]. Ovaries, second only to the thyroid gland, express significant levels of the sodium–iodine symporter gene, leading to iodine accumulation in the ovaries [126]. Studies on radioactive iodine treatment indicate that small and growing follicles absorb more iodine, crucial for ovarian granulosa cell secretory activities [128]. Estrogen influences iodine absorption by the ovaries, promoting iodine accumulation in large Graafian follicles [126, 129].

Mild to moderate iodine deficiency is prevalent among reproductive-aged women globally [130, 131]. Iodine is abundant in fish, poultry, and seaweed and is also available as a dietary supplement [129]. Evidence suggests that iodine supplementation of 150 μg/day should begin preconception, as starting during pregnancy may be too late [98].

### 2.15. Copper and Female Reproductive Health

Copper is an essential micronutrient crucial for fetal growth and development, playing a pivotal role in energy supply, myelination, neurotransmitter metabolism, and connective tissue development [132]. Research shows that infertile women often have lower copper intake compared to control groups, with a notable correlation between dietary antioxidant intake and infertility risk [133]. Elevated follicular fluid copper levels are associated with improved oocyte growth, fertilization, and embryo differentiation, underscoring copper's significance in reproductive outcomes [134]. Reduced serum copper levels have also been observed in women experiencing miscarriage [132, 135].

The relationship between copper metabolism and sex hormones highlights copper's importance in female reproductive health. Estradiol upregulates copper transporter expression, facilitating copper handling and impacting the efficacy of copper supplementation [136]. Copper interacts directly with gonadotropin-releasing hormone (GnRH), enhancing LH release and influencing ovarian function [136]. Excessive estrogen production can alter copper distribution, affecting hormonal balance and reproductive function.

Studies in mice have linked Cu, Zn-superoxide dismutase (Cu, Zn-SOD) deficiency with impaired progesterone secretion and infertility [137]. Reduced plasma copper levels in infertile women may disrupt the structural integrity and function of supporting collagen within the Graafian follicle, and diminished plasma copper could hinder ovum transport through the fallopian tubes [138, 139]. Elevated serum copper has been implicated in unexplained infertility, with studies showing higher levels in patients with PCOS compared to fertile counterparts [140, 141]. Excess copper may suppress progesterone levels, leading to anovulation, implantation failure, or luteal phase deficits, and can impede the absorption of essential minerals like zinc [142].

Copper-rich foods include organ meats, seafood, nuts, and seeds [129]. The RDA for adults aged 19 years and older is 900 μg daily for women. Evaluating serum copper levels is essential, and dietary adjustments or supplements may be beneficial in managing infertility related to micronutrient imbalances.

### 2.16. Vitamin C and Female Reproductive Health

Vitamin C, or ascorbic acid, is a water-soluble antioxidant and essential cofactor in hydroxylation and amidation reactions [143]. It plays a significant role in collagen synthesis, proteoglycans, and the intercellular matrix [144]. High concentrations of vitamin C are found in seminal plasma, which may prevent DNA damage, and in ovarian tissue, suggesting an active transport mechanism into follicular fluid during folliculogenesis [145–147]. The ovary accumulates and turns over ascorbic acid, with the highest levels in the theca interna, granulosa, and luteal compartments [148, 149].

Research indicates that increased ascorbic acid intake enhances tissue inhibiting metalloproteinases, thereby improving follicle survival. Additionally, supplementation of ascorbic acid can elevate serum progesterone levels in individuals with luteal phase defects, potentially ameliorating these conditions [150, 151]. Ascorbic acid deficiency is linked to ovarian atrophy, extensive follicular atresia, and premature resumption of meiosis [152].

Dietary supplementation with vitamin C during pregnancy is recommended to reduce the incidence of birth defects, with a suggested daily intake of at least 500 mg starting as early as possible during pregnancy [153, 154].

### 2.17. Zinc and Female Reproductive Health

Zinc, an essential mineral found in foods and supplements, plays crucial roles in protein and DNA synthesis, immune function, and cell division [155]. It acts as an antioxidant by scavenging superoxide anions, although high supplementation levels can induce mitochondrial OS [156]. Adequate zinc levels are vital for oocyte quality, maturation, fertilization, and implantation. Zinc influences various stages of fertility, including ovulation and the menstrual cycle, and is essential for DNA synthesis critical to oocyte development [3, 157]. It is integral to gene transcription, protein synthesis, and numerous cellular processes, exhibiting both antioxidant and pro-oxidant actions [158, 159].

Dietary factors largely determine zinc status, and additional zinc is recommended during pregnancy to support maternal and fetal needs [160]. Many women in developed countries meet their zinc requirements through multivitamin supplements [119]. While limited randomized controlled trials (RCTs) have been conducted, evidence suggests that micronutrient supplementation, including zinc, may positively affect female fertility [119, 161]. Multivitamin and multimineral (MMN) supplementation in women with fertility problems can normalize trace element levels, potentially enhancing the quality of the microfollicular environment and improving reproductive outcomes [161–163]. During pregnancy, it is recommended that women consume 11 mg of zinc daily [164].

### 2.18. Vitamin B12 and Female Reproductive Health

Also known as cobalamin, vitamin B12 is a water-soluble vitamin that serves as a cofactor in DNA synthesis, fatty acid, and amino acid metabolism. It plays a crucial role in one-carbon metabolism as an enzymatic cofactor [165]. Deficiency in vitamin B12 can potentially influence reproductive outcomes by affecting reproductive hormones [166]. Riboflavin, a precursor to flavin mononucleotide and flavin adenine dinucleotide, interacts with enzymes crucial to cholesterol synthesis and sex-steroid hormone metabolism [166]. Insufficient levels of vitamin B12 are prevalent among infertile women, impacting ovarian responsiveness and pregnancy outcomes [167].

Studies indicate associations between key players in one-carbon metabolism, such as riboflavin, vitamin B-6, vitamin B-9, and vitamin B-12, with ovarian responsiveness and pregnancy outcomes. Higher plasma homocysteine levels, a marker of impaired one-carbon metabolism, increase the risk of anovulation [123]. Recent analyses suggest an inverse association between the intake of riboflavin, vitamin B-6, and vitamin B-12 and the risk of ovulatory infertility [161]. Additionally, deficiencies in both RBC folate and vitamin B12 are prevalent among infertile women, potentially impacting embryo viability [167, 168]. The recommended daily intake of B12 during pregnancy is 2.6 μg [164, 169].

### 2.19. Manganese and Female Reproductive Health

Found naturally in foods, manganese acts as a cofactor for numerous enzymes and plays crucial roles in various metabolic pathways, including amino acid metabolism, cholesterol synthesis, and immunological responses [170, 171]. It is essential for female reproduction and fetal development, although excessive levels can have toxic effects [132, 172]. Deficiencies or excesses of essential trace elements, including manganese, are associated with female infertility and adverse pregnancy outcomes [173, 174]. Previous studies indicate that women requiring assisted reproductive technologies often exhibit lower levels of essential trace elements [174]. Maternal micronutrient deficiency may increase the risk of metabolic disorders in offspring, and deficiencies in essential trace elements may adversely affect both maternal and fetal health [173]. This underscores the importance of personalized assessment and improvement of trace element status in improving female reproductive health. Both positive and negative manganese balances were observed during late pregnancy in women with a daily manganese intake averaging between 2 and 7 mg [169].

## 3. Conclusion

Infertility poses significant challenges not only for affected individuals and couples but also for society at large, impacting family structures and public health systems. Given the complex interplay of various lifestyle factors influencing fertility, optimal preconception nutrition, particularly the intake of essential micronutrients, emerges as a critical area of focus. These key micronutrients and their specific roles in female reproductive health are summarized in Table 2, providing a concise overview of their physiological significance and clinical relevance.

This review underscores the pivotal role of specific vitamins and minerals such as folate, vitamin D, iron, and antioxidants in enhancing reproductive health. These micronutrients are vital for numerous physiological processes including oocyte quality, hormonal balance, and the establishment of a healthy pregnancy. The evidence suggests that many women of reproductive age, particularly those experiencing infertility, may not meet the recommended dietary allowances for these nutrients, which could adversely affect their fertility potential.

Considering these findings, it is crucial to establish standardized guidelines for micronutrient supplementation tailored to women planning for pregnancy. Healthcare providers should prioritize nutritional counseling as part of preconception care, ensuring that women receive personalized assessments of their dietary intake and micronutrient status. This proactive approach can empower women to make informed dietary choices that may enhance their fertility outcomes.

## Figures and Tables

**Table 1:** Research strategy.

Objects	Specification
Source of research	PubMed, Google Scholar, ScienceDirect, and hand search in the web base

Key terms (including MeSH terms and free text search terms)	Micronutrients, trace elements, dietary supplements, infertility, female, and fertility
	• Due to the variety of paper issues, after finding papers with key terms, papers related to our aim of this research included in the final step.

Timeframe	Publication between 2000 and April 2024 (articles and data before 2000 were limited and not suitable for our title).

Inclusion and exclusion criteria	Inclusion criteria: all kinds of studies about micronutrients and trace elements and their effects on women's fertility (only English-language articles, full-text availability, preceding, abstracts, decertations, letters, commentaries, and reviews).
	Exclusion criteria: non-English and unpublished studies.

**Table 2:** Summary of key micronutrients in female reproductive health.

Nutrient	Role in reproduction	Effects of deficiency	Recommended daily intake	Food sources	Special considerations
Folate (vitamin B9)	DNA synthesis, egg quality, and embryo development	Increased anovulation, reduced IVF success, and miscarriages	0.4 mg/day preconception and 0.6 mg in pregnancy	Green leafy vegetables, beans, eggs, and fruits	Higher doses (1–4 mg) for women at high risk of NTDs
Selenium	Antioxidant balance, follicular environment, and gametogenesis	Pregnancy complications and infertility	40 μg/day preconception and 60 μg in pregnancy	Brazil nuts, seafood, and eggs	Both low and high plasma levels are associated with low pregnancy rates
Nickel	Impacts endocrine glands, reproductive hormones, and oxidative stress	Reduced fertility	No established RDA	Diverse food and drinking water	Higher absorption on an empty stomach, effects on fertility need more study
Vitamin A	Antioxidant, influences follicular growth, steroidogenesis, oocyte quality	Oxidative stress reduces oocyte quality	5000 IU (upper limit)	Vegetables, dairy products, eggs, and fruits	Avoid high doses due to teratogenic effects
Vitamin B-6	Homocysteine metabolism, impacts on oocyte and embryo quality	Hyperhomocysteinemia, recurrent miscarriages	1.9 mg/day during pregnancy	Beef liver, tuna, salmon, fortified cereals, poultry, bananas, and leafy greens	Higher intake needed during pregnancy
Iodine	Thyroid hormone synthesis, promotes ovulation	Delayed conception, miscarriage, ovarian cysts	150 μg/day preconception	Fish, poultry, and seaweed	Supplementation should start preconception
Copper	Energy supply, influences sex hormones and GnRH activity	Infertility, miscarriage	900 μg/day	Organ meats, seafood, nuts, and seeds	Imbalance can affect fertility; excessive levels may suppress progesterone
Vitamin C	Antioxidant, collagen synthesis, enhances follicle survival	Ovarian atrophy, follicular atresia	500 mg/day	Citrus fruits, strawberries, bell peppers, and broccoli	High concentration in ovarian tissue, supplementation during pregnancy recommended
Zinc	DNA synthesis, immune function, influences oocyte quality and maturation	Impaired oocyte development, anovulation	11 mg/day during pregnancy	Meat, shellfish, legumes, seeds, and nuts	High doses can cause oxidative stress
Vitamin B12	DNA synthesis, impacts on reproductive hormones	Infertility, impaired embryo viability	2.6 μg/day during pregnancy	Meat, fish, dairy products, and fortified cereals	Deficiency prevalent among infertile women
Manganese	Amino acid metabolism, cholesterol synthesis, immune responses	Infertility, adverse pregnancy outcomes	2–7 mg/day	Nuts, whole grains, and leafy vegetables	Both deficiencies and excesses can be harmful
L-Arginine	Improves endometrial receptivity, enhances implantation rates, influences ovarian function and follicular development	Increased risk of implantation failure, decreased ovarian function	Optimal dosage undetermined, varies between 6 g/day and 16 g/day	Seafood, nuts, seeds, meats, and soy proteins	Further research is needed on optimal dosage
Vitamin E	Antioxidant, increases anti-Mullerian hormone (AMH) levels, antral follicle count (AFC), and mean ovarian volume (MOV)	Increased oxidative stress, reduced AMH levels, compromised follicle count	Recommended daily allowance (RDA) 15 mg/day	Almonds, vegetable oils, and grains	Monitor for potential interactions with other supplements
Vitamin D	Regulates gene expression in reproductive tissues, improves oocyte activation, maturation, and IVF outcomes, enhances fertility by increasing antioxidant levels	Increased risk of infertility, compromised oocyte quality	Recommended daily intake around 15 μg/day	Fish, mushrooms, cod liver oil, cheese, beef liver, and eggs	Supplement if dietary intake is inadequate
Omega-3 fatty acids	Enhances folliculogenesis, oocyte maturation, embryo quality, and implantation, influences prostaglandin biosynthesis	Reduced fertility, compromised oocyte and embryo quality	Optimal intake varies; supplementation may be beneficial	Nuts and fish	Consider individual dietary needs
L-carnitine	Improves oxidative and metabolic health, enhances endometrial receptivity, implantation rates, and live birth rates	Reduced endometrial receptivity, implantation failure	Optimal dosage around 3 g/day	Meat, fish, and some other animal products	Further research needed on optimal dosage
Beta-carotene	Enhances follicular development, oocyte quality, and ovarian function, acts as an antioxidant	Increased oxidative stress, compromised folliculogenesis	Optimal intake undetermined	Many fruits and vegetables	Consider individual dietary needs
Coenzyme Q10 (CoQ10)	Functions as an antioxidant, supports ATP synthesis, improves clinical pregnancy rates (CPR), enhances gamete physiology	Increased oxidative stress, reduced ATP production	Recommended daily doses range from 5 to 30 mg/kg	Meat, fish, nuts, and some oils	Monitor for potential side effects
*N*-Acetylcysteine	Acts as a potent antioxidant, regulates luteinizing hormone (LH), follicle-stimulating hormone (FSH), and testosterone levels, improves oocyte quality	Increased oxidative stress, hormonal imbalances	Optimal dosage around 1200–1800 mg/day	Cysteine in chicken, turkey, yogurt, cheese, eggs, sunflower seeds, and legumes	Consult with healthcare provider

## Data Availability

Data sharing is not applicable to this article as no new data were created or analyzed in this study.
